# Normoglycemic High Anion Gap Metabolic Acidosis With Hyperkalemia: An Unusual Presentation of Alcoholic Ketoacidosis

**DOI:** 10.7759/cureus.110494

**Published:** 2026-06-08

**Authors:** Armaan Mishra, Mohammad Shagil, Lakhan Singh

**Affiliations:** 1 Internal Medicine, Rajmata Shrimati Devendra Kumari Singhdeo Government Medical College Ambikapur, Ambikapur, IND; 2 General Medicine, Chhattisgarh Institute of Medical Sciences, Bilaspur, IND

**Keywords:** alcoholic ketoacidosis, diabetic ketoacidosis, hyperkalemia, metabolic acidosis, starvation ketosis

## Abstract

Alcoholic ketoacidosis (AKA) is a form of high anion gap metabolic acidosis characterized by ketosis, typically occurring in malnourished individuals after binge alcohol consumption. If not identified early, it can lead to life-threatening electrolyte disturbances and cardiac arrhythmias.

A man in his fifties presented with altered mental status and shortness of breath. Arterial blood gas analysis revealed severe metabolic acidosis with a pH of 7.22, a bicarbonate level of 4.2 mmol/L, and normal lactate levels. Urine ketones were strongly positive (4+). Electrocardiography demonstrated tall T waves, and serum potassium was elevated at 5.8 mEq/L. The combination of recent binge alcohol use, normoglycemia, ketosis, and high anion gap metabolic acidosis suggested AKA. Prompt fluid resuscitation and electrolyte correction resulted in progressive clinical improvement, and the patient was discharged without complications.

This case highlights the importance of considering AKA in normoglycemic patients presenting with high anion gap metabolic acidosis and ketosis, particularly those with a history of excessive alcohol consumption. Accurate differentiation from diabetic ketoacidosis is essential because management strategies differ significantly. It is further distinguished by significant hyperkalemia with electrocardiographic changes, an atypical presentation of AKA in which serum potassium levels are usually normal or low.

## Introduction

Alcoholic ketoacidosis (AKA), a clinical condition primarily associated with chronic alcoholism, is characterized by high anion gap metabolic acidosis. It commonly presents with nausea, vomiting, abdominal pain, and altered sensorium, often in the setting of normoglycemia. Because its presentation may mimic diabetic ketoacidosis (DKA) and other causes of severe metabolic acidosis, delayed recognition can result in significant morbidity and potentially life-threatening cardiac complications [1,2].

Although total body potassium depletion is common in AKA, serum potassium levels are usually normal or low at presentation due to chronic malnutrition and gastrointestinal losses. Significant hyperkalemia with electrocardiographic changes is uncommon and may create diagnostic uncertainty during initial evaluation. We report a case of AKA presenting with severe metabolic acidosis and hyperkalemia with tall T waves, highlighting an important diagnostic pitfall and the need for early differentiation from DKA to guide appropriate management.

## Case presentation

A male patient in his fifties presented with altered sensorium and shortness of breath after poor oral intake for four days. He had a history of chronic alcohol use and had reportedly lost consciousness for approximately 30 minutes following a recent episode of binge drinking. Although a history of excessive alcohol consumption was corroborated by family members, the exact daily alcohol intake could not be reliably quantified. On presentation, his Glasgow Coma Scale score was 10/15 (E2V4M4).

Initial vital signs revealed a blood pressure of 80/60 mmHg, heart rate of 130 beats/min, respiratory rate of 20 breaths/min, and oxygen saturation of 77% on room air, which improved to 98% with oxygen delivered via a plain face mask at 5 L/min. Random blood glucose was 139 mg/dL. Electrocardiography demonstrated generalized tall, peaked T waves (Figure 1), and serum potassium was elevated at 5.8 mEq/L (reference range: 3.5-5.0 mEq/L).

**Figure 1:**
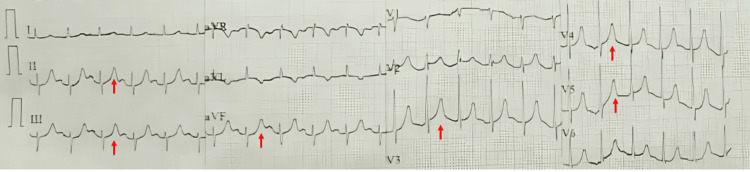
Twelve-Lead ECG Twelve-lead electrocardiogram showing generalized tall, peaked T waves (red arrows) across limb and precordial leads consistent with hyperkalemic changes.

In view of hyperkalemia with electrocardiographic changes, the patient was treated with intravenous calcium gluconate (10 mL of 10% solution over 10 minutes), insulin-dextrose therapy, sodium bicarbonate, and nebulized albuterol.

Initial hematological and biochemical investigations revealed leukocytosis, thrombocytopenia, acute kidney injury, and marked ketonuria (Table 1). In view of the leukocytosis and hemodynamic instability at presentation, an evaluation for infectious etiologies was undertaken. The Widal test, peripheral smear for malarial parasites, dengue NS1 antigen, dengue IgM/IgG, and ELISA testing for leptospirosis and scrub typhus were all negative.

**Table 1:** Laboratory Parameters at Presentation

Laboratory Parameter	Patient’s Value	Reference Range
Hemoglobin (g/dL)	13.5	12.0-18.0
White blood cell count (/mm^3^)	21,000	4000-11000
Platelet count (/mm^3^)	121,000	150,000-300,000
Blood urea (mg/dL)	67	10-50
Creatinine (mg/dL)	1.9	0.6-1.4
Serum sodium (mEq/L)	134.4	136-147
Serum potassium (mEq/L)	5.8	3.5-5
Serum chloride (mEq/L)	109.7	98-106
HbA1c (%)	5.6	4.5-6.5
Urine ketones	4+	Negative

The patient's history was negative for illicit substance use; however, a formal toxicology screen was not performed. Despite initial fluid resuscitation with a balanced crystalloid solution (Sterofundin), hypotension persisted, necessitating transfer to the intensive care unit for norepinephrine support.

Arterial blood gas analysis demonstrated severe acidemia with a mixed metabolic acidosis consisting of high anion gap and hyperchloremic metabolic acidosis (anion gap 20.5 mmol/L) (Table 2). Urine ketones were strongly positive (4+). Although DKA was initially considered, the combination of normoglycemia, normal HbA1c (5.6%), recent binge alcohol consumption, and marked ketonuria favored a diagnosis of AKA.

**Table 2:** Arterial Blood Gas Parameters at Presentation and After 24 Hours of Treatment

Parameter	At Presentation	After 24 Hours	Reference Range
pH	7.22	7.43	7.35–7.45
PaCO2 (mm Hg)	10.1	21.7	35-45
PaO2 (mm Hg)	113.2	106.2	80-100
Bicarbonate (mmol/L)	4.2	14.7	22-26
Lactate (mmol/L)	0.74	0.98	0.5-2.2
Anion gap	20.5	15.0	8-16

Management included continued resuscitation with a balanced crystalloid solution (Sterofundin), followed by 5% dextrose-containing fluids, thiamine, multivitamin supplementation, and supportive care. The patient required norepinephrine support because of persistent hypotension despite initial fluid resuscitation. With correction of volume depletion and metabolic abnormalities, his hemodynamic status gradually improved, allowing tapering and discontinuation of norepinephrine.

Serial laboratory assessments demonstrated progressive improvement in acid-base status (Table 2), with pH increasing from 7.22 to 7.43 and bicarbonate from 4.2 mmol/L to 14.7 mmol/L after 24 hours of treatment. Serum potassium normalized to 4.6 mEq/L, accompanied by resolution of electrocardiographic abnormalities and progressive clinical improvement.

The patient remained hospitalized for seven days, including four days in the intensive care unit followed by three days in the general ward. Early recognition and timely correction of metabolic and electrolyte abnormalities resulted in sustained clinical and biochemical recovery, allowing discharge without complications.

## Discussion

AKA should be considered in patients presenting with high anion gap metabolic acidosis and ketosis, particularly in the setting of recent binge alcohol consumption, chronic alcohol dependence, or poor nutritional intake [1]. Patients with AKA typically present with nonspecific symptoms such as nausea, vomiting, abdominal pain, and varying degrees of altered mental status. Signs of volume depletion, including tachycardia and hypotension, are common and may be accompanied by manifestations of alcohol withdrawal [3]. In the present case, altered sensorium, persistent hypotension, severe high anion gap metabolic acidosis, and marked ketonuria were the predominant presenting features.

The diagnosis of AKA is primarily clinical and is supported by the presence of metabolic acidosis, ketosis, and a history of recent alcohol consumption. Our patient demonstrated severe acidemia (pH 7.22), markedly reduced bicarbonate levels (4.2 mmol/L), an elevated anion gap (20.5 mmol/L), and strongly positive urine ketones while maintaining normoglycemia and a normal HbA1c level. The disproportionate reduction in bicarbonate relative to the increase in anion gap suggested a concomitant hyperchloremic metabolic acidosis in addition to the high anion gap acidosis. These findings, together with a history of recent binge alcohol intake, strongly supported the diagnosis of AKA.

The principal differential diagnoses in patients presenting with ketosis and metabolic acidosis include DKA, starvation ketosis, and AKA. DKA was initially considered because of the severe metabolic acidosis and ketonuria; however, normoglycemia and a normal HbA1c favored AKA. Early differentiation between these two conditions is crucial, as inappropriate insulin administration in patients with AKA may precipitate severe hypoglycemia [4]. The metabolic acidosis in AKA is primarily driven by ketogenesis, with predominant production of β-hydroxybutyrate in the setting of recent alcohol consumption and poor nutritional intake (Figure 2).

**Figure 2:**
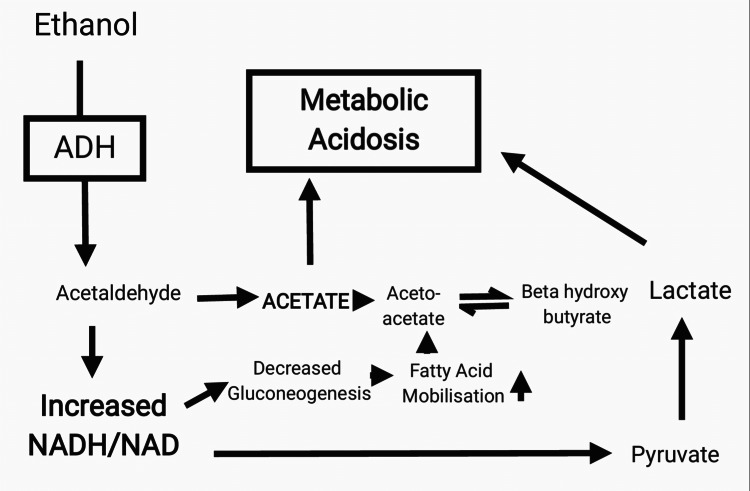
Metabolic Pathways Involved in Alcoholic Ketoacidosis Schematic illustration showing ethanol metabolism, alterations in the NADH/NAD⁺ ratio, ketone body production, and the resulting development of metabolic acidosis. ADH, alcohol dehydrogenase; NAD⁺, nicotinamide adenine dinucleotide (oxidized); NADH, nicotinamide adenine dinucleotide (reduced).

Compared with patients with DKA, individuals with AKA typically exhibit higher β-hydroxybutyrate-to-acetoacetate and lactate-to-pyruvate ratios [5].

Starvation ketosis was considered in the differential diagnosis; however, it is generally associated with less severe metabolic acidosis than that seen in the present case. Furthermore, acute ethanol intoxication alone rarely results in significant lactic acidosis or ketoacidosis in the presence of adequate caloric intake, suggesting that the combination of recent binge alcohol consumption and poor nutritional status played a key role in the development of AKA in our patient [6].

Lactic acidosis secondary to sepsis or tissue hypoperfusion was considered a potential contributor to the metabolic acidosis, given the patient's hypotension, leukocytosis, and hypoxemia at presentation. However, the normal serum lactate level (0.74 mmol/L), rapid improvement with supportive therapy, and negative infectious workup made this diagnosis unlikely. Toxic alcohol ingestion, including methanol and ethylene glycol poisoning, may also present with high anion gap metabolic acidosis but is typically not associated with significant ketosis. Careful evaluation of the clinical history, biochemical findings, and acid-base status is therefore essential to avoid diagnostic errors and ensure appropriate management.

A notable feature of this case was significant hyperkalemia (5.8 mEq/L) with tall T waves on electrocardiography. While serum potassium levels in AKA are typically normal or low, our patient developed marked hyperkalemia, likely due to severe acidemia, acute kidney injury, and transcellular potassium shifts. This atypical finding increased diagnostic complexity and the risk of cardiac arrhythmias.

Management of AKA includes volume resuscitation, thiamine supplementation, correction of metabolic abnormalities, nutritional support, and close monitoring. Thiamine should be administered before dextrose-containing fluids to support cellular metabolism and reduce the risk of Wernicke encephalopathy [2,7]. In the present case, initial resuscitation was performed with a balanced crystalloid solution (Sterofundin), followed by 5% dextrose to suppress ketogenesis and restore carbohydrate availability [8,9]. Thiamine, multivitamin supplementation, and supportive care were also provided to address nutritional deficiencies associated with chronic alcohol use.

Hyperkalemia was managed with intravenous calcium gluconate, insulin-dextrose therapy, sodium bicarbonate and nebulized albuterol. Correction of volume depletion, acidemia, and electrolyte abnormalities resulted in resolution of ketosis and progressive clinical improvement.

Long-term management should focus on alcohol cessation, nutritional rehabilitation, and psychosocial support. Strong family and social support are essential for maintaining abstinence and reducing the risk of recurrence [10,11].

## Conclusions

AKA should be considered in normoglycemic patients presenting with high anion gap metabolic acidosis, ketosis, and a history of recent alcohol consumption. This case highlights significant hyperkalemia with electrocardiographic changes as an atypical presentation that may increase diagnostic complexity and the risk of cardiac complications. Early recognition, differentiation from DKA, and prompt initiation of fluid resuscitation, electrolyte correction, thiamine supplementation, and nutritional support are essential for favorable outcomes.
